# Optimising Generalisable Deep Learning Models for CT Coronary Segmentation: A Multifactorial Evaluation

**DOI:** 10.1007/s10278-025-01677-2

**Published:** 2025-09-18

**Authors:** Shisheng Zhang, Ramtin Gharleghi, Sonit Singh, Chi Shen, Dona Adikari, Mingzi Zhang, Daniel Moses, Dominic Vickers, Arcot Sowmya, Susann Beier

**Affiliations:** 1 School of Mechanical and Manufacturing Engineering, University of New South Wales, Sydney, Australia; 2 School of Computer Science and Engineering, University of New South Wales, Sydney, Australia; 3 School of Medicine, University of New South Wales, Sydney, Australia; 4 Centre for Healthy Futures, Torrens University Australia, Sydney, NSW Australia; 5 Department of Radiology, Prince of Wales Hospital, Sydney, Australia; 6 Department of Cardiology, Prince of Wales Hospital, Sydney, Australia

**Keywords:** Convolutional neural networks, Coronary artery segmentation, Deep learning, Model generalisability, Computed tomography coronary angiography, CTCA

## Abstract

Coronary artery disease (CAD) remains a leading cause of morbidity and mortality worldwide, with incidence rates continuing to rise. Automated coronary artery medical image segmentation can ultimately improve CAD management by enabling more advanced and efficient diagnostic assessments. Deep learning-based segmentation methods have shown significant promise and offered higher accuracy while reducing reliance on manual inputs. However, achieving consistent performance across diverse datasets remains a persistent challenge due to substantial variability in imaging protocols, equipment and patient-specific factors, such as signal intensities, anatomical differences and disease severity. This study investigates the influence of image quality and resolution, governed by vessel size and common disease characteristics that introduce artefacts, such as calcification, on coronary artery segmentation accuracy in computed tomography coronary angiography (CTCA). Two datasets were utilised for model training and validation, including the publicly available ASOCA dataset (40 cases) and a GeoCAD dataset (70 cases) with more cases of coronary disease. Coronary artery segmentations were generated using three deep learning frameworks/architectures: default U-Net, Swin-UNETR, and EfficientNet-LinkNet. The impact of various factors on model generalisation was evaluated, focusing on imaging characteristics (contrast-to-noise ratio, artery contrast enhancement, and edge sharpness) and the extent of calcification at both the coronary tree and individual vessel branch levels. The calcification ranges considered were 0 (no calcification), 1–99 (low), 100–399 (moderate), and > 400 (high). The findings demonstrated that image features, including artery contrast enhancement (*r* = 0.408, *p* < 0.001) and edge sharpness (*r* = 0.239, *p* = 0.046), were significantly correlated with improved segmentation performance in test cases. Regardless of severity, calcification had a negative impact on segmentation accuracy, with low calcification affecting the segmentation most poorly (*p* < 0.05). This may be because smaller calcified lesions produce less distinct contrast against the bright lumen, making it harder for the model to accurately identify and segment these lesions. Additionally, in males, a larger diameter of the first obtuse marginal branch (OM1) (*p* = 0.036) was associated with improved segmentation performance for OM1. Similarly, in females, larger diameters of left main (LM) coronary artery (*p* = 0.008) and right coronary artery (RCA) (*p* < 0.001) were associated with better segmentation performance for LM and RCA, respectively. These findings emphasise the importance of accounting for imaging characteristics and anatomical variability when developing generalisable deep learning models for coronary artery segmentation. Unlike previous studies, which broadly acknowledge the role of image quality in segmentation, our work quantitatively demonstrates the extent to which contrast enhancement, edge sharpness, calcification and vessel diameter impact segmentation performance, offering a data-driven foundation for model adaptation strategies. Potential improvements include optimising pre-segmentation imaging (e.g. ensuring adequate edge sharpness in low-contrast regions) and developing algorithms to address vessel-specific challenges, such as improving segmentation of low-level calcifications and accurately identifying LM, RCA and OM1 of smaller diameters.

## Introduction

Coronary artery disease (CAD) is a leading cause of mortality and morbidity worldwide, posing a significant public health challenge [1]. Non-invasive imaging techniques such as computed tomography coronary angiography (CTCA) play a critical role in visualising the coronary arteries and cardiac tissue, aiding in the detection and management of CAD [2]. Accurate segmentation of the coronary arteries from CTCA scans is a key step in emerging research, such as delineating artery lumen to enable anatomical measurement and blood flow analysis [3–7]. Recent advancements in deep learning technologies drive the development of algorithms capable of automatically segmenting coronary arteries from CTCA with high accuracy [3]. Despite the great potential of deep learning-based segmentation methods to streamline research workflows and ultimately advance diagnostic capacity, training such models still requires significant manual or semi-automatic annotations for testing, validation and verification. The annotation of private datasets can be extremely time-consuming and resource intensive. Therefore, researchers continuously seek methods to leverage pre-annotated public datasets for model training and subsequent application to unannotated private datasets [8]. However, the accuracy of deep learning-based segmentation models trained on open-source datasets often declines when applied to private datasets due to the variability in datasets [9], differences in image characteristics due to differing imaging protocols and equipment used across different organisations, varying signal intensities across patients, and patient-specific features such as disease severity and different ethnic populations [10]. Disease severity, particularly the presence of calcification, significantly impacts segmentation performance. Calcification associated with atherosclerotic plaque has been identified as one of the primary sources of error in coronary artery segmentation using CTCA, as calcified lesions cause blooming artifacts [11–15], leading to higher lumen intensities in affected areas [16]. These findings confirm and align with our focus in this paper, demonstrating that calcifications consistently compromise segmentation accuracy across imaging techniques. Previous studies have not examined the extent to which different levels of calcification affect segmentation performance. Investigating this aspect could provide a more targeted approach to model adaptation for varying disease distributions.

The anatomical structure of coronary arteries varies significantly [17], even across populations, influencing both imaging characteristics and segmentation accuracy. For example, studies have shown that the dimensions of coronary artery segments in Indian populations are smaller compared to those reported in studies from other continents [18, 19]. Additionally, while coronary arteries are usually surrounded by a layer of fat, in some individuals, they may be embedded directly within the cardiac muscle, which affects their anatomical context [20]. Yet, no study has specifically identified the geometric variations in coronary artery structure that are most likely to contribute to decreased segmentation performance of models across datasets. Addressing this gap is crucial for improving model generalisability and ensuring reliable segmentation performance in diverse clinical settings.

While previous studies have investigated imaging protocols and their effects [21, 22], it remains unclear whether these specific parameters have been systematically assessed in relation to model performance across different datasets. Several studies have explored individual aspects of image quality in medical segmentation tasks. For instance, one study measured contrast-to-noise ratio and demonstrated that denoising in CT images can enhance vascular structure segmentation [23]. Similarly, a study proposed a pre-filtering method for images with low contrast-to-noise ratio, showing that this approach significantly improved liver vessel segmentation in 3D CT angiography images [24]. Another study investigated varying levels of contrast enhancement and their impact on coronary artery centreline extraction, a commonly performed analysis task, and found that improved contrast enhancement led to better extraction performance [25]. Additionally, research has suggested that due to the injection of contrast material, artery voxels exhibit significant intensity changes over time, emphasising that contrast enhancement depends on the time interval between contrast injection and imaging, which varies across clinical settings [26]. Improved edge sharpness has also been shown to be beneficial for various segmentation tasks. For example, a specialised Sharpness-Aware model was developed for retinal vessel segmentation, demonstrating its effectiveness in improving segmentation accuracy [27]. Another study found that edge sharpness plays a crucial role in brain ventricle segmentation [28]. Despite these studies, there is still limited research evaluating the effects of contrast-to-noise ratio, artery contrast enhancement, and edge sharpness on deep learning-based models for coronary artery segmentation across different datasets.

In addition to investigations of image characteristics, several deep learning-based approaches have been proposed to improve segmentation generalisability. For example, in contrast-enhanced cardiac Magnetic Resonance Imaging (MRI) [29] and cardiac cine MRI [30], researchers have demonstrated that data augmentation strategies can enhance segmentation robustness across clinical centres. Additionally, a meta-learning framework was introduced to identify representations associated with domain shifts between source and unseen datasets, improving generalisation in low-data settings [31]. While these strategies highlight progress in other imaging modalities, their application to CTCA remains relatively limited, particularly as the specific factors influencing segmentation performance in CTCA have not yet been systematically characterised.

Here we investigate how specific image factors and specific coronary artery shape contribute to the generalisability of segmentation models for coronary arteries using CTCA scans. This work examines image properties, including contrast-to-noise ratio, artery contrast enhancement, and edge sharpness, as potential factors that may affect model generalisability. Furthermore, we consider patient-specific factors such as the geometry of coronary arteries (mean absolute curvature and mean diameter) [32] and the severity of calcification in coronary arteries. We assess segmentation performance using the dice similarity coefficient across two distinct datasets, first training and validating deep learning models on the ASOCA public dataset [33–35], and then testing them on a private dataset GeoCAD to evaluate model generalisability. Rather than proposing a new model architecture, this study aims to systematically assess the generalisability of existing deep learning models across datasets with different image characteristics, anatomical features, and calcification severity. This aspect of model evaluation remains underexplored in the current literature.

## Materials and Methods

### CTCA Datasets and Coronary Arteries

The study utilised two distinct datasets: the publicly accessible ASOCA dataset [33–35] and the GeoCAD dataset [36], collected separately at Spectrum Medical Imaging, Sydney, Australia. Ethics obtained for ASOCA was approved by the University of New South Wales Human Research Ethics Committee (Ref. 022961). Ethics obtained for GeoCAD was approved by the St Vincent’s Hospital Human Research Ethics Committee, Sydney (Ref. 2020/ETH02127) and the NSW Population and Health Service Research Ethics Committee (Ref. 2021/ETH00990). The NSW Ministry of Health approved this work for publication per relevant regulations.

The ASOCA dataset comprises 40 CTCA cases, of which 20 were from normal individuals without stenosis/non-obstructive coronary arterial disease and 20 from patients with obstructive disease with evidence of calcium scores greater than 0 [33–35]. Image acquisition was performed with a retrospective ECG-gated multi-detector CT scanner (GE Lightspeed 64 multi-slice scanner), where the 64-slice configuration results in more limited temporal resolution compared to modern scanners. To achieve optimal imaging conditions, beta-blockers were administered to maintain a heart rate of 60 bpm or lower during the intravenous contrast-enhanced scans (Omnipaque 350). The collected images were stored as DICOM files, focusing on the end-diastolic phase for subsequent analysis. The z-axis resolution was consistently 0.625 mm, whereas the in-plane resolution fluctuated between 0.3 mm and 0.4 mm, subject to individual patient variability and the Field of View selected.

The private GeoCAD dataset [36] was created from a retrospective cohort study that included 450 adults undergone CTCA imaging due to suspected CAD. The dataset was collected from multiple centres using a variety of CT scanners (including models from GE and Siemens) and generally prospective ECG-gated imaging. Compared to ASOCA, GeoCAD has more diverse cases, including patients with narrowed arteries, varying circulation dominance, stents or bypass grafting. A random GeoCAD subset of 70 cases was annotated by two experts and subsequently validated by a senior expert. Each GeoCAD case is accompanied by a coronary artery calcium score and information on the distribution of calcified plaques across coronary arteries. Higher scores suggest more significant calcification, causing an imaging artefact called “blooming”, a well-known challenge for segmentation. This subset of 70 annotated cases can be subdivided into four categories, stratified by varying coronary artery calcium scores (0, 1–99, 100–399, > 400), as shown in Table 1 [37].

**Table 1:** Datasets statistics. ASOCA dataset is for model training and validation. GeoCAD dataset is used for model testing. CAC score refers to the coronary artery calcium score

Datasets	Size	Accessibility	Patient characteristics
Training and validation			
ASOCA (total)	40	Public	50% normal and 50% diseased
No calcification	20		CAC score = 0
Low calcification	8		1 ≤ CAC score ≤ 99
Moderate calcification	6		100 ≤ CAC score ≤ 399
High calcification	6		CAC score ≥ 400
Sex			
Male	25		
Female	15		
Testing			
GeoCAD (total)	70	Private	Patients with various degrees of calcification
No calcification	12		CAC score = 0
Low calcification	29		1 ≤ CAC score ≤ 99
Moderate calcification	21		100 ≤ CAC score ≤ 399
High calcification	8		CAC score ≥ 400
Sex			
Male	32		
Female	38		

Additional image characteristics were derived from the ASOCA and GeoCAD datasets, employing Nibabel [38] and NumPy [39] for data extraction, including image shape, voxel dimension, contrast-to-noise ratio, artery contrast enhancement, and edge sharpness [40–42]. The image shapes and voxel dimensions are displayed in coronal, sagittal, and axial directions in Table 2.

**Table 2:** Image properties of CTCA datasets

Image properties	Plane	ASOCA	GeoCAD
Matrix dimension (min–max)	Coronal Sagittal Axial	(512–512) (512–512) (168–224)	(512–512) (512–512), (192–464)
Voxel dimension (mm, min–max)	Coronal Sagittal Axial	(0.306–0.494) (0.306–0.494) (0.625–0.625)	(0.332–0.588) (0.332–0.588) (0.500–0.625)

The formulas used for contrast-to-noise ratio, artery contrast enhancement, and edge sharpness are specified in Algorithms 1–3. The contrast-to-noise ratio measures the contrast of the artery against the background noise. High artery contrast enhancement suggests adequate contrast agent uptake, making the coronary arteries more delineated for more effective diagnosis [40, 41]. Artery contrast enhancement is estimated by comparing the mean attenuation within the artery to that of surrounding non-arterial tissue. Although not a comparison between contrast-enhanced and non-contrast images, it serves as a practical proxy for assessing contrast clarity, which is particularly relevant for deep learning-based segmentation tasks that rely on visual distinction of arterial structures within the anatomical context. A high degree of edge sharpness indicates a substantial distinction between the arteries and the background, essential for precisely identifying arterial features. The attenuation gradient at the arterial boundary evaluated this parameter [42].

**Algorithm 1:**
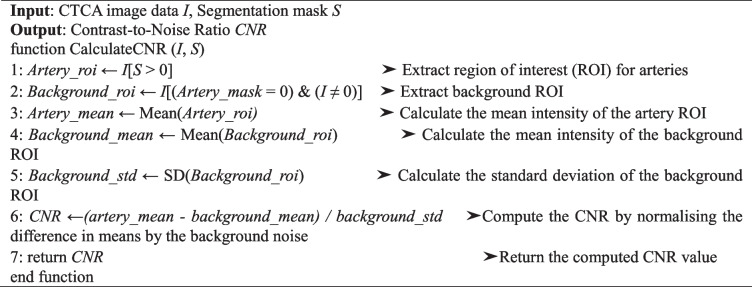
Calculate contrast-to-noise ratio in CTCA images

**Algorithm 2:**
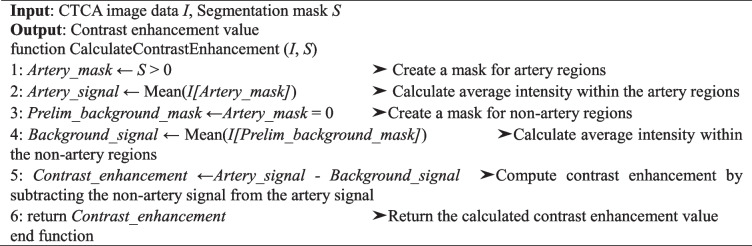
Calculate artery contrast enhancement in CTCA images

**Algorithm 3:**
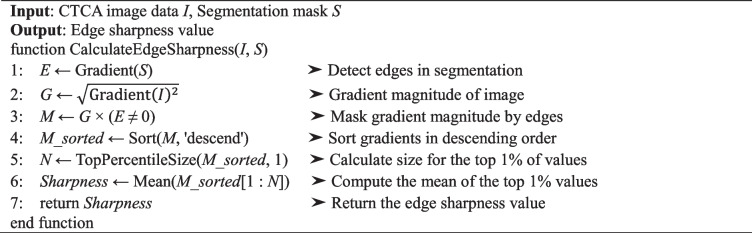
Calculate edge sharpness in CTCA images

**Fig. 1:**
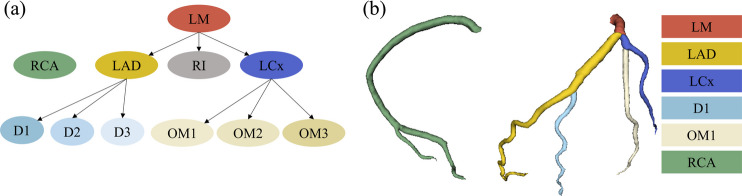
Coronary artery anatomy and the specific arterial branches analysed in this study. **a** A network diagram of the coronary artery tree including the left main coronary artery (LM), left anterior descending artery (LAD) with its diagonal branches (D1-D3), left circumflex artery (LCx) with its obtuse marginal branches (OM1-OM3), right coronary artery (RCA), and the ramus intermedius (RI). **b** Three-dimensional representation of coronary artery tree and artery segments selected for this study

Within the scope of both datasets, coronary artery segmentation was carried out in line with coronary anatomy (Fig. 1a). The coronary arterial structure arises from the aorta into two primary branches: the left and right coronary arterial trees. On the left, the major branches of clinical importance are the left main coronary artery (LM), which usually bifurcates into the left anterior descending artery (LAD) and the left circumflex artery (LCx), with the addition of a trifurcation which includes the ramus intermedius artery in some instances [43]. The LAD typically features the first diagonal artery (D1) as its first major branch, while the left circumflex’s first significant branch is the first obtuse marginal (OM1). Typically, the diameter of the coronary arteries narrows as the arteries extend further from the aorta, often accompanied by changes in curvature. In contrast, the right coronary artery (RCA) exhibits a distinct anatomical structure, predominantly supplying blood to the right side of the heart through its proximal and distal branches. Illustrated in Fig. 1b are the essential segments selected for this study: the LM, LAD, LCx, the first diagonal branch (D1), the first obtuse marginal branch (OM1), and the RCA. The segments were extracted from the coronary artery tree using VMTK branch extractor [44] and labelled manually based on the anatomy. The maximum inscribed sphere radius and mean curvatures were calculated based on VMTK extracted centreline.

### Coronary Artery Segmentation

We analysed the factors affecting the generalisability of deep learning models for CTCA segmentation using (i) nnU-Net [45], (ii) Swin-UNETR [46], and (iii) EfficientNet-LinkNet [47], which are described below. The reason for choosing deep learning over other methods is its growing popularity in both medical imaging research and industry for segmentation tasks. These three architectures/frameworks were selected because they represent the latest advancements in deep learning-based architectures for coronary artery segmentation.

(i) The U-Net has established itself as a highly effective architecture for medical image segmentation due to its encoder-decoder structure [48]. Built upon U-Net, the nnU-Net framework has demonstrated its capability through successful applications in various medical imaging tasks [45]. The nnU-Net leverages common rules of thumb to train a U-Net model without manual hyperparameter tuning. These settings enable the nnU-Net to adaptively configure a basic U-Net for better segmentation accuracy tailored to the specific dataset it encounters.
(ii) The second deep learning architecture we evaluated is the Swin-UNETR [46], which combines the Swin Transformer’s capabilities with the proven U-Net design. As traditional Vision Transformers face limitations, such as the memory requirements growing rapidly, Swin-UNETR addresses these by limiting long-range interactions to save on memory, employing a local attention mechanism to process complex patterns by capturing multi-scale details [46].
(iii) The third architecture in our comparative analysis is the EfficientNet-LinkNet architecture, which was identified as a promising model through comprehensive evaluation of 25 distinct encoder-decoder architectures [47]. EfficientNet provides an efficient and scalable backbone, balancing the depth, width, and resolution of the network through a compound scaling method. When coupled with the LinkNet decoder, the resulting architecture showed high accuracy on segmentation tasks.

### Model Training, Validation, and Analysis Methods

Three segmentation models were trained and validated using CTCA scans from the ASOCA dataset, as described in the “Coronary Artery Segmentation” section. After that, the models were tested on the GeoCAD dataset to determine their performance and the ability to generalise across a new dataset. In particular, the nnU-Net architecture was subjected to a training procedure using the 3D full-resolution default configurations [45]. The decision to use the default settings was motivated by the intention to evaluate the model’s default capabilities without additional parameter tuning, providing a baseline for its ability. Swin UNETR was trained using the AdamW optimiser, a variant of the Adam optimiser that incorporates a decoupled weight decay regularisation [49]. The training and validation details of EfficientNet-LinkNet can be found elsewhere [47]. All networks were trained using fivefold cross-validation. After training, the fivefold cross-validation outputs were merged separately for each model: nnU-Net segmentations were combined using soft ensembling, while segmentations from Swin-UNETR and EfficientNet-LinkNet were merged using majority voting [50]. Finally, the outputs from all three models (nnU-Net, Swin-UNETR, and EfficientNet-LinkNet) were merged using majority voting to produce the final segmentation. This approach leverages the strengths of multiple models while reducing the impact of their individual weaknesses. Ensembling combines outputs from multiple models, and helps ensure that observed performance variations are less likely to be due to a single model’s weaknesses. The coronary artery tree and artery-wise segmentation were evaluated using the dice similarity coefficient (DSC). For the analysis of coronary artery trees, the correlations between DSC and image properties (contrast-to-noise ratio, artery contrast enhancement, and edge sharpness) were quantitatively assessed using the Pearson correlation coefficient [51]. Statistical significance for the associations between these variables was established at a *p*-value threshold of less than 0.05. For the analysis of the characteristics of coronary artery segments, the relationship between DSC and artery segment geometries (using VMTK commands, as described in the “CTCA Datasets and Coronary Arteries” section) was also quantitatively assessed using the Pearson correlation coefficient [51]. The statistical analyses were performed using Python v3.10 and relevant libraries, including NumPy, Pandas, and SciPy for data manipulation and statistical calculations, and Matplotlib for visualisation.

## Results

### Image Characteristics and Coronary Artery Tree Segmentation

**Fig. 2:**
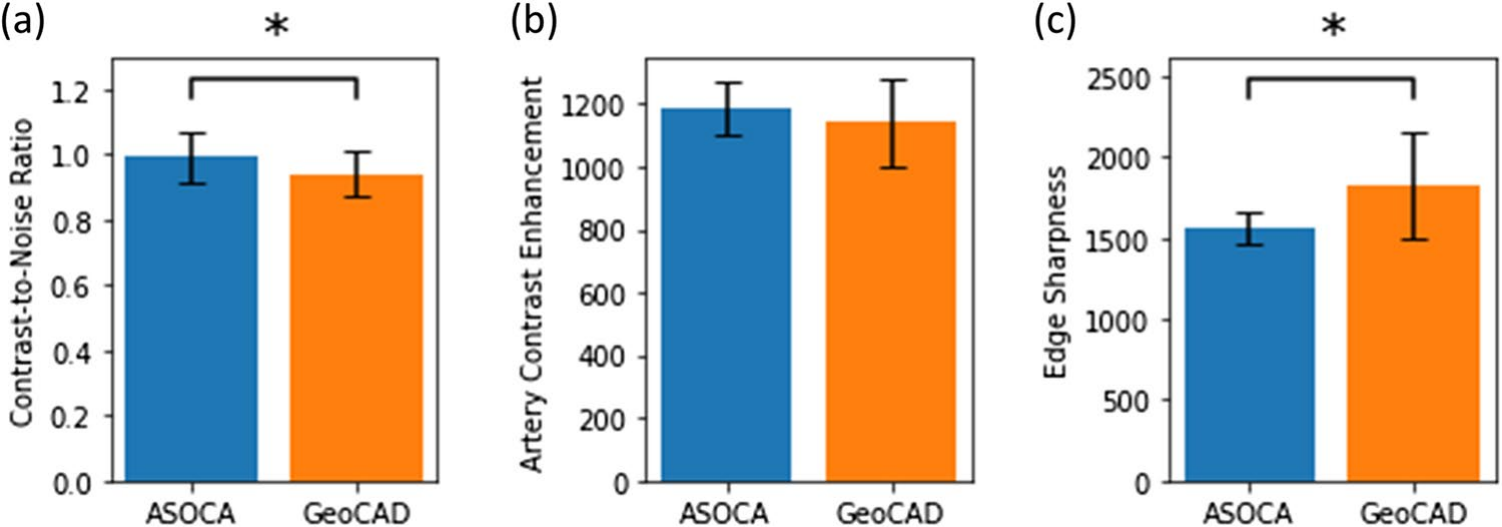
Image quality metrics were assessed to compare the visual quality of coronary artery tree segmentation between ASOCA and GeoCAD datasets. Contrast-to-Noise Ratio (**a**), artery contrast enhancement (**b**), and edge sharpness (**c**)

We evaluated the segmentation performance on the coronary artery tree in ASOCA and GeoCAD using DSC. As expected, ASOCA showed a slight advantage with a score of around 0.9, while GeoCAD scored approximately 0.8. This is because the models were trained and evaluated using fivefold cross-validation on ASOCA. The significant difference indicates that using the trained model to predict GeoCAD leads to lower segmentation performance. Our comparative analysis examined the difference in three image quality metrics: contrast-to-noise ratio, artery contrast enhancement, and edge sharpness between datasets (Fig. 2). Overall, we found significant differences in all metrics except for artery contrast enhancement. This suggests that both ASOCA and GeoCAD have similar enhanced arterial CTCA contrast.

To further explore the factors affecting segmentation performance, we investigated the correlation between DSC and the three image quality metrics within GeoCAD, the test dataset (Table 3). The analysis focused on understanding how variations in image characteristics influence model accuracy. Specifically, artery contrast enhancement exhibited a strong positive correlation with DSC (Pearson correlation coefficient *r* = 0.408, *p*-value < 0.001), indicating that higher artery contrast enhancement is associated with higher DSC values. Similarly, edge sharpness was positively correlated with DSC (*r* = 0.239, *p* = 0.046), suggesting that sharper edges contribute to increased DSC. While the contrast-to-noise ratio also showed a positive correlation with the DSC (*r* = 0.201), this relationship was not statistically significant (*p* = 0.095).

**Table 3:** Correlation of dice similarity coefficient (DSC) with contrast-to-noise ratio, artery contrast enhancement and edge sharpness on GeoCAD dataset

Image characteristics	Pearson correlation coefficient	*p*-value
Contrast-to-noise ratio	0.201	0.095
Artery contrast enhancement	0.408	< 0.001*
Edge sharpness	0.239	0.046*

### Diseases and Coronary Artery Tree Segmentation

We evaluated the impact of calcification on coronary artery tree segmentation performance by comparing DSC between the ASOCA and GeoCAD datasets across different calcification levels (Table 4). When tested on a holdout set from the ASOCA dataset, the deep learning models demonstrated high segmentation accuracy, achieving DSCs ranging from 0.85 ± 0.10 to 0.90 ± 0.03 across calcification levels. However, when tested on the external GeoCAD dataset, performance decreased slightly in groups with less calcification. Specifically, the DSC dropped from 0.90 ± 0.03 to 0.82 ± 0.07 in the No Calcification group and from 0.86 ± 0.03 to 0.79 ± 0.09 in the low calcification group.

**Table 4:** Dice similarity coefficient (DSC ± SD) of coronary artery tree segmentation of ASOCA and GeoCAD datasets across calcification levels

Calcification level	ASOCA (DSC ± SD)	GeoCAD (DSC ± SD)	*p*-value
No Calcification	0.90 ± 0.03	0.82 ± 0.07	0.001*
Low Calcification	0.86 ± 0.03	0.79 ± 0.09	0.006*
Moderate Calcification	0.87 ± 0.04	0.81 ± 0.09	0.055
High Calcification	0.85 ± 0.10	0.82 ± 0.07	0.548

Welch’s *t*-test was conducted for each calcification level to assess statistical significance between the ASOCA and GeoCAD groups. Statistically significant differences (*p* < 0.05) were found in the no and low calcification levels, while differences in the moderate and high calcification levels were not statistically significant. These findings suggest that segmentation performance is more sensitive to subtle calcifications when tested on an unseen dataset. Such regions may be more difficult to detect and segment due to their smaller, less distinct deposits compared to moderate and high calcification. These subtle features are more likely to be obscured by contrast and challenging for the models to detect and exclude accurately. Additionally, the GeoCAD dataset was acquired using more modern scanners with reduced motion artifacts. This mismatch in acquisition quality could further impact the model’s ability to generalise, particularly for smaller calcified plaques, which are more sensitive to motion artifacts.

### Coronary Artery Segment Analysis

**Fig. 3:**
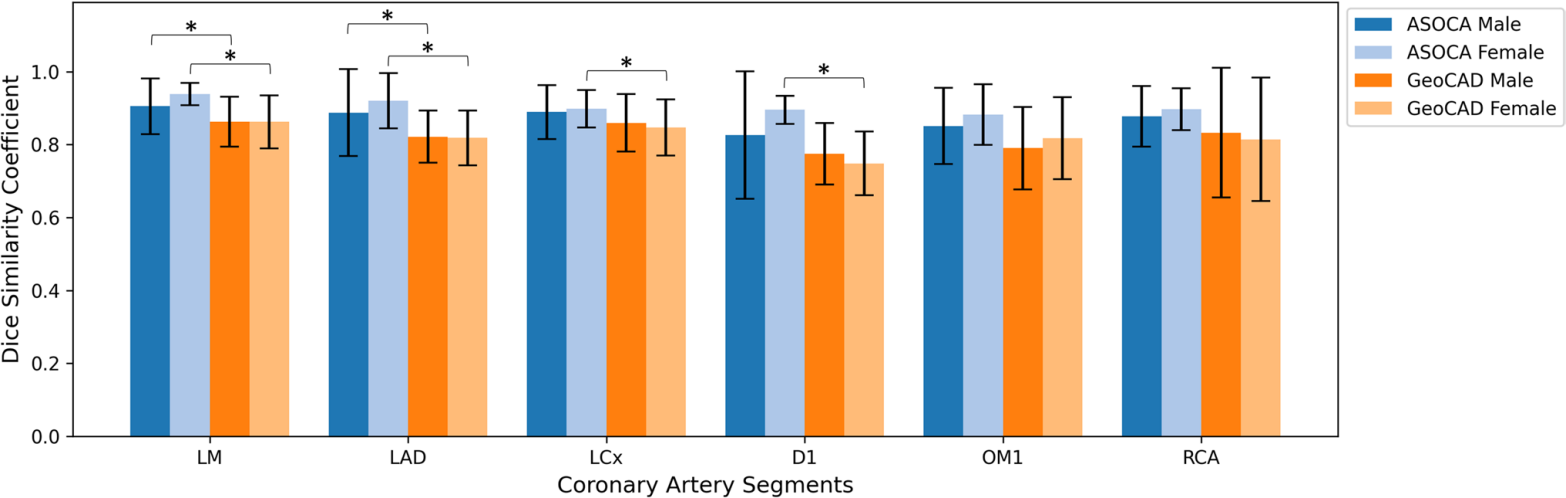
Dice similarity coefficient of coronary artery segments

We further assessed the segmentation accuracy of models trained and validated on the ASOCA dataset and subsequently tested on the GeoCAD dataset, by focusing on various coronary artery segments: the left main (LM), left anterior descending (LAD), left circumflex (LCx), the first diagonal branch (D1), the first obtuse marginal branch (OM1), and the right coronary artery (RCA), as shown in Fig. 3. To account for known gender-related differences in coronary artery anatomy [52, 53], particularly the generally smaller artery diameters observed in female subjects, we stratified the analysis by sex. All segment-wise performance comparisons were conducted within gender groups (i.e. ASOCA males were compared with GeoCAD males and ASOCA females with GeoCAD females). As mentioned in the “Materials and Methods” section, after obtaining the initial artery segmentation from the CTCA image, it was converted to a mesh to facilitate processing in VMTK. Vessel splitting was performed at the mesh level using VMTK. The split mesh was then used to map and identify the included points back into the original CTCA segmentation domain, ensuring that DSCs are calculated accurately within the binary image context.

This comparative analysis aimed to identify the impact of anatomical variations on the test performance of deep learning models. Our findings revealed that when applied to the GeoCAD test dataset, the models’ segmentation DSCs were sensitive to the anatomical characteristics of the different artery segments. As shown in Fig. 3, segmentation models trained and validated on the ASOCA dataset generally performed better on ASOCA subjects (Fig. 3, blue columns) than on those from GeoCAD (Fig. 3, orange columns) across both male and female groups. This performance gap is most noticeable in the LM, LAD, LCx (which are crucial for supplying oxygenated blood to major heart regions), and D1 segments, where DSCs are significantly lower in the GeoCAD group, especially for females. Although OM1 and RCA segments did not show significant differences between datasets, the overall trend across segments highlights the challenge of generalising model performance to external datasets, particularly when anatomical variations are present. This result suggests that segment-specific and sex-specific anatomical differences can substantially influence segmentation accuracy.

**Fig. 4:**
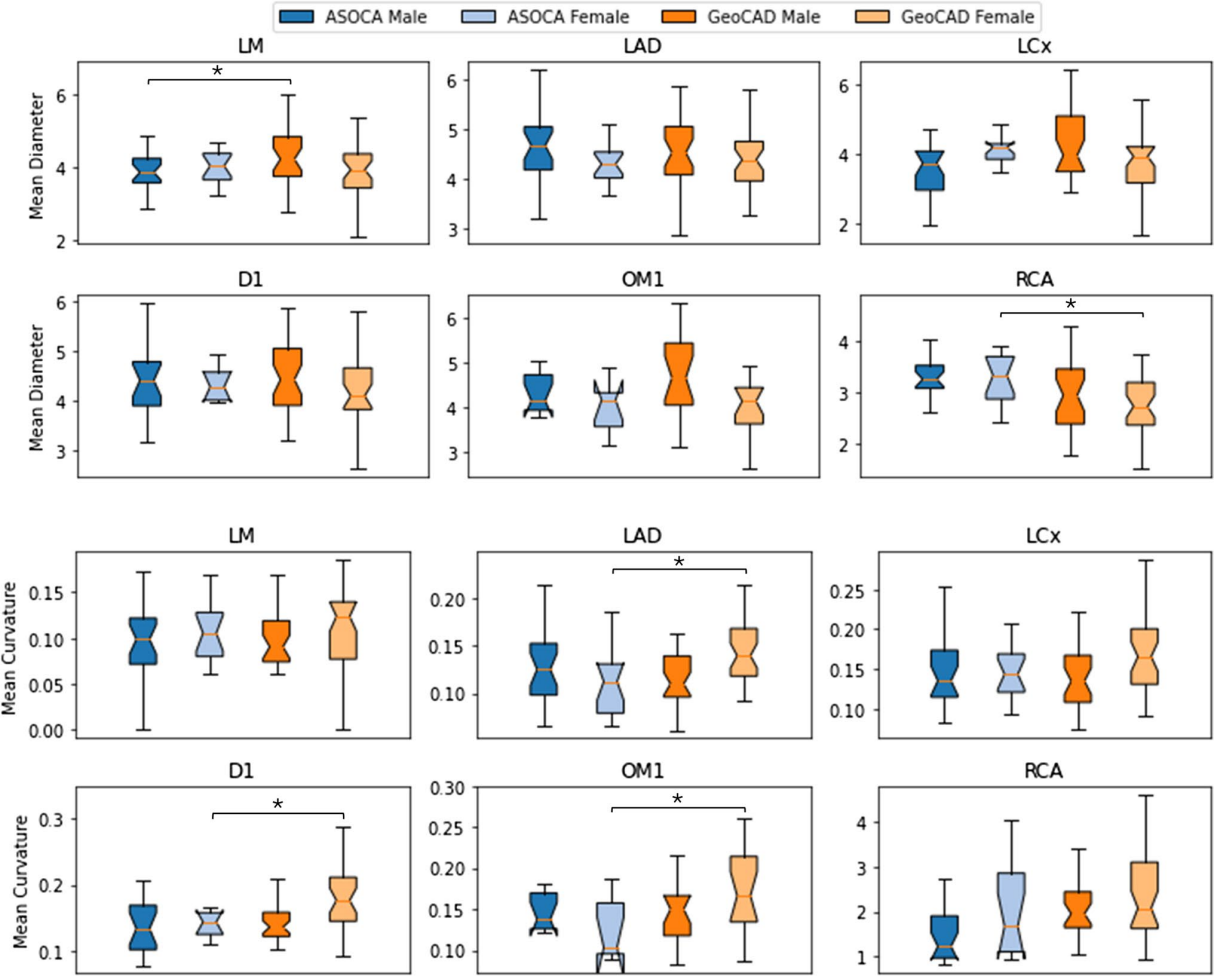
Mean diameter (top two rows) and curvature (bottom two rows) of coronary artery segments. LM: left main coronary artery, LAD: left anterior descending artery, LCx: left circumflex artery, D1: first diagonal branch, OM1: first obtuse marginal branch, RCA: right coronary artery

We analysed arterial geometry based on the ground truth annotations to further explore anatomical differences across the datasets. Both ASOCA and GeoCAD showed broadly similar mean artery diameters across segments, with substantial overlap in their interquartile ranges (Fig. 4). Female subjects in both datasets had consistently smaller diameters and higher curvatures. However, diameter distributions in GeoCAD were more diverse. Curvature differences between ASOCA and GeoCAD were more pronounced than diameter differences. Significant curvature variation was observed in the female LAD (*p* < 0.05), D1 (*p* < 0.05), and OM1 (*p* < 0.05), with GeoCAD exhibiting a greater variability and higher median curvature. These differences in geometry align with the segment-wise performance decline (Fig. 3).

The correlation analysis on the GeoCAD dataset, based on ground truth annotations, offers an understanding of how anatomical features, specifically the mean diameter and curvature of coronary artery segments, influence the accuracy of segmentation models (Table 5). In the LM segment of female subjects, a positive correlation between LM diameter and LM DSC (coefficient, 0.429; *p*-value, 0.008) suggests that larger diameters are associated with higher DSC in this segment. However, LM curvature showed minimal influence on LM DSC, suggesting it is less critical for segmentation accuracy in the LM segment. Conversely, both diameter and curvature exhibited weak correlations with DSC for the LAD segment, indicating that these geometrical features have little to no impact on segmentation accuracy. The LCx segment showed a weak positive correlation between diameter and DSC, hinting at a potential influence of diameter size on segmentation accuracy. The curvature within this segment displayed a negative correlation with DSC. OM1 of male subjects (coefficient, 0.470; *p*-value, 0.036) and RCA of female subjects (coefficient, 0.640; *p*-value, < 0.001) both demonstrated a strong positive correlation between diameter and DSC, reinforcing the idea that larger arterial diameters contribute positively to segmentation accuracy. However, the curvature in these segments seemed to have a negligible impact on the DSC, indicating that it might not be as crucial a factor for segmentation accuracy in these areas.

**Table 5:** Correlation analysis of dice similarity coefficient, mean diameter, and curvature across anatomical segments on GeoCAD dataset. An asterisk (*) indicates *p* < 0.05

Artery segment	Male				Female			
	Diameter		Curvature		Diameter		Curvature	
	Coefficient	*p*-value	Coefficient	*p*-value	Coefficient	*p*-value	Coefficient	*p*-value
LM	0.111	0.552	− 0.025	0.894	0.429	0.008*	0.181	0.283
LAD	− 0.009	0.962	− 0.093	0.620	0.205	0.231	0.071	0.682
LCx	0.234	0.205	0.106	0.572	0.196	0.259	− 0.110	0.529
D1	0.073	0.669	− 0.132	0.438	− 0.151	0.418	− 0.288	0.116
OM1	0.470	0.036*	− 0.178	0.452	0.469	0.050	0.128	0.614
RCA	0.072	0.711	0.047	0.807	0.640	< 0.001*	0.068	0.688

## Discussion

The generalisability of deep learning-based segmentation models is crucial, particularly in the context of coronary artery analysis, where public datasets are limited. This paper investigates the factors influencing test performance and the differences between the ASOCA and GeoCAD cohorts, which serve as a foundation for this exploration. The ASOCA dataset, publicly accessible and characterised by an equal distribution of normal and diseased individuals, offers a controlled environment for training deep learning models. However, decreased performance emerges when the models are tested against the GeoCAD dataset, which includes more diseased cases and presents greater challenges for segmentation.

For non-contrast imaging modalities, several studies have explored how deep learning models trained on a single data source fail to generalise effectively when applied to unseen imaging domains, such as new clinical centres or different imaging protocols [54, 55]. For example, a recent challenge at MICCAI 2020 on cardiac cine-MRI segmentation demonstrated that models trained in single-centre, single-vendor settings often require additional adaptation to maintain performance in new domains [56]. Various strategies have been proposed to address, including extensive spatial and intensity-based data augmentation, domain adaptation techniques, and transfer learning, enabling models to adapt their learned representations to unseen imaging sources. Additionally, synthetic data generation using generative models and meta-learning approaches has been investigated to improve model robustness across diverse datasets [30, 31].

Despite progress in non-contrast imaging, limited work has addressed generalisation in contrast-enhanced imaging, which introduces additional complexities. Using contrast agents leads to intensity heterogeneity and imaging artifacts, which alter data distributions and complicate the generalisation of models to new imaging domains. Moreover, variations in imaging protocols, particularly in the timing between contrast agent injection and image acquisition, result in substantial differences in image appearance across centres [57]. Consequently, solutions that have proven effective for non-contrast imaging do not directly transfer to contrast-enhanced imaging, highlighting the need for further investigation into specialised generalisation techniques for this domain.

Initial studies in this area have shown promising results. Several works have demonstrated that a combination of data augmentation, transfer learning, and domain adaptation can significantly improve the generalisability of single-domain models in contrast-enhanced imaging [29]. These findings suggest that models trained in single-centre settings, when enriched with suitable generalisation strategies, can achieve robust performance comparable to or even exceeding that of multi-centre models. This is particularly important in contrast-enhanced imaging, where the availability of large, interoperable datasets remains limited. However, previous studies have still highlighted the need for further research to understand the impact of imaging protocol variations and intensity heterogeneity on model performance, which is a focus of this study.

Prior research has shown that image quality factors significantly influence segmentation accuracy across various imaging tasks [23–28]. However, their specific impact on coronary artery segmentation has not been systematically assessed. One of the key findings of this study is the significant contribution of artery contrast enhancement and edge sharpness to segmentation performance, as indicated by their positive correlation with segmentation performance measured by DSC. High artery contrast enhancement reflects adequate contrast agent uptake, which enhances the visibility of coronary arteries and facilitates more effective diagnosis [40, 41]. Similarly, a high degree of edge sharpness ensures a clear distinction between the arteries and surrounding structures, enabling precise identification of arterial boundary [42]. Both image characteristics are critical for achieving accurate segmentation outcomes, emphasising the importance of maintaining high-quality imaging protocols to ensure consistent model performance on new, unseen datasets. However, it is important to note that these image quality metrics are also influenced by the underlying ground truth annotations. Differences in annotation strategies may have contributed to observed variations in metric values, in addition to inherent differences in image characteristics. Building on these observations, it is important to note that artery contrast enhancement and edge sharpness are influenced by CTCA acquisition parameters, such as contrast injection protocols and scanner reconstruction algorithms [58, 59]. Although artery contrast enhancement and edge sharpness were quantified post-acquisition using segmentation masks in this study, they reflect imaging characteristics that can be modified during image acquisition and through post-processing. For example, using sharper, edge-enhancing reconstruction kernels and motion-compensated acquisition techniques can improve artery boundary visibility during scanning [60, 61]. In parallel, post-acquisition strategies such as adaptive histogram equalisation can help align artery contrast enhancement and edge sharpness distributions between source and target datasets [62], thereby reducing domain shift and improving model generalisability. These findings suggest that artery contrast enhancement and edge sharpness are useful retrospectively and can also inform prospective imaging protocol design. In this context, it is also worth noting that the GeoCAD dataset, which exhibits higher mean edge sharpness than ASOCA, was acquired using prospective ECG-gated CT imaging. This technique reduces motion artefacts by capturing images during a stable phase of the cardiac cycle, resulting in improved arterial boundary. In contrast, the ASOCA dataset was collected using retrospective gating, which may contribute to its comparatively lower edge sharpness. Although the training and testing configuration was not reversed in this study, such an experiment could further clarify how differences in image quality influence model generalisability. In addition to the metrics used in this study, no-reference image quality assessment methods such as BRISQUE [63] and DistilIQA [64] have shown promise in general CT image evaluation. Although not yet fully validated for CTCA, adapting these approaches may offer complementary insights in future work.

Furthermore, segmentation models often struggle with generalisation due to variations in patient-specific factors such as disease severity and ethnicity [9, 10]. Calcification remains a major source of segmentation errors, as previously demonstrated in both CTCA and intravascular ultrasound studies, where signal attenuation caused by calcified regions leads to misclassification and reduced accuracy [65, 66]. However, the extent to which different calcification levels affect segmentation performance remains underexplored. We found that the calcification level significantly affects DSC, primarily due to “bloom artifacts” in the images (Table 4). These artifacts complicate segmentation: small calcifications blend with the surrounding lumen, where contrasted blood flows, making them difficult to distinguish. Conversely, large calcifications overly brighten the surrounding area, obscuring lumen boundaries. The transition from the ASOCA cohort, which consists of 50% diseased and 50% non-diseased cases, to the all-disease context of GeoCAD, combined with the pronounced artifacts of all calcification levels as indicated by Welch’s *t*-test statistics, demonstrate the negative effect of calcification on segmentation performance. Segmentation performance in the validation set (ASOCA) remained relatively consistent across calcification levels. However, in the test set (GeoCAD), a statistically significant drop in DSC was observed for the no-calcification and low-calcification groups, while the moderate and high calcification groups did not show significant differences. Although this overall decline in performance may be partially explained by overfitting, the specific drop in the low-calcification group may reflect challenges in distinguishing small calcified lesions from the contrast-enhanced lumen. Additionally, other factors such as anatomical variability or lower image contrast may also contribute to reduced segmentation accuracy in low and no-calcification cases. Particularly, the group of low calcification exhibits the most reduction in DSC compared to the ASOCA cohort, which is likely due to the fact that low calcification areas are smaller than moderate and high calcification, making them less likely to be accurately excluded from the blood domain during segmentation process. Since the contrast medium enhances the lumen, it appears bright on CTCA scans. Lumen intensities are higher in the parts with highly calcified lesions due to blooming artifacts. Therefore, for highly calcified lesions, intensity cut-off values are locally compensated by subtracting a percentage of the difference to correct for the cut-off values in these locations. However, the subtle difference in intensity between low-calcification lesions and the contrast-enhanced lumen may not be effectively recognised by the model, leading to segmentation inaccuracies. These findings highlight a key limitation in accurately segmenting arteries near low-calcification lesions. To improve segmentation performance across datasets, an approach that explicitly accounts for calcium levels as a matching criterion could enhance the transferability of segmentation algorithms.

Additionally, anatomical variability, including differences in coronary artery structure and surrounding tissues across populations, may influence segmentation outcomes, however specific geometric features contributing to performance drops remain unidentified [20]. This study highlights the importance of anatomical variations in model generalisability. For instance, a larger coronary artery diameter significantly correlates with increased accuracy in segmenting LM, OM1, and RCA arteries. The impact of curvature is less predictable, with only a weak negative correlation with D1 in females. Moreover, the consistently lower accuracy in segmenting the D1 and OM1 arteries across both datasets may be attributed to the varied distribution of contrast with blood flow, which occurs at bifurcations where only a fraction of blood enters these branches. In this case, it may lead to lower contrast, and lower brightness, making it challenging to segment arteries. Furthermore, anatomical variations between genders may contribute to challenges in predicting coronary artery segmentation, as females are known to have smaller artery diameters and higher curvature typically [67, 68]. Beyond gender-related differences, broader anatomical variability is a critical factor influencing segmentation performance and generalisability, particularly in CTCA where artery geometry, branching patterns, and spatial orientation can differ substantially between individuals. Addressing this challenge requires a data-driven approach to dataset development. Expanding datasets to include scans from multiple clinical centres, diverse patient demographics, and a wide range of disease presentations can help include more anatomical variation. In addition, synthetic data augmentation methods, including geometry-based modifications [69], simulation of rare anatomical topology [70], and artificial generation of calcified plaques [71], may further enhance the diversity and representativeness of the training set. These strategies contribute to the development of generalisable segmentation models. We would like to note that this analysis is intended to be exploratory in nature. As such, no correction for multiple comparisons was applied. The goal is to identify potential trends and contributing factors that may influence segmentation performance, with the reported associations serving as a basis for future hypothesis-driven investigations rather than confirmed conclusions. In this study, we used 15 females and 25 males for training from ASOCA dataset, while the test set, GeoCAD, had 38 females and 32 males. To minimise the confounding effect of gender on segmentation performance, we grouped subjects by gender and analysed the geometric impact on DSC separately. Since female subjects tend to have smaller diameters, training specialised models to process small artery diameter in female patients may have a more significant positive effect on DSC compared to male patients. Therefore, in order to generalise the segmentation model across multiple datasets, certain preprocessing methods are necessary, including the following: (i) adapting artery contrast enhancement and edge sharpness from target CTCA to source CTCA, (ii) employing techniques to process bloom artifacts caused by calcified plaque, and (iii) developing methods to account for variations in artery geometry between populations. Additionally, generating synthetic datasets that incorporate these variations could be valuable in improving model robustness and ensuring better generalisation across diverse clinical datasets.

A key limitation of this study is the relatively small sample size, particularly for stratified subgroup analyses. While the number of cases was sufficient for initial hypothesis generation and consistent trends were observed, larger annotated datasets would enhance statistical power and support more detailed modelling. In addition, formal assessments of repeatability and inter-reader agreement were not included. Although each case was independently annotated by two experts and subsequently validated by a senior expert for consistency, future work should incorporate formal inter-reader validation to further support reproducibility. Finally, as this study is exploratory in nature, the findings suggest that accounting for imaging characteristics and anatomical variability may help improve segmentation generalisability. However, additional validation using larger and more diverse datasets is required to determine whether addressing these specific factors will consistently lead to more generalisable models.

## Conclusion

In summary, enhancing the generalisability of coronary artery segmentation models requires consideration of imaging quality, disease-related artifacts and anatomical variations in new, unseen datasets. Our findings include a positive correlation between artery contrast enhancement, edge sharpness and the dice similarity coefficients in test cases. Also, while all levels of calcification negatively affect the dice similarity coefficients, low calcification presents the statistically significant decrease. Furthermore, the diameters of specific artery branches show significant correlations with the dice similarity coefficients. Addressing differences between datasets, particularly concerning image and patient characteristics, is essential for advancing the capabilities of deep learning-based segmentation models in this domain.

## Data Availability

This study uses two datasets. The ASOCA dataset is publicly available at https://asoca.grand-challenge.org/. The GeoCAD dataset is an internal resource and is not publicly available.
